# The Digital Divide and Seeking Health Information on Smartphones in Asia: Survey Study of Ten Countries

**DOI:** 10.2196/24086

**Published:** 2022-01-13

**Authors:** Xiaohui Wang, Jingyuan Shi, Kwan Min Lee

**Affiliations:** 1 Department of Media and Communication City University of Hong Kong Hong Kong Hong Kong; 2 Department of Communication Studies Hong Kong Baptist University Hong Kong Hong Kong; 3 Wee Kim Wee School of Communication and Information Nanyang Technological University Singapore Singapore

**Keywords:** smartphone, health information seeking, Asia, user profile, digital divide

## Abstract

**Background:**

Although recent developments in mobile health have elevated the importance of how smartphones empower individuals to seek health information, research investigating this phenomenon in Asian countries has been rare.

**Objective:**

The goal of our study was to provide a comprehensive profile of mobile health information seekers and to examine the individual- and country-level digital divide in Asia.

**Methods:**

With survey data from 10 Asian countries (N=9086), we ran multilevel regression models to assess the effect of sociodemographic factors, technological factors, and country-level disparities on using smartphones to seek health information.

**Results:**

Respondents who were women (*β*=.13, *P*<.001), parents (*β*=.16, *P*<.001), employed (*β*=.08, *P*=.002), of higher social status (*β*=.08, *P*<.001), and/or from countries with low health expenditures (*β*=.19, *P*=.02) were more likely to use smartphones to seek health information. In terms of technological factors, technology innovativeness (*β*=.10, *P*<.001) and frequency of smartphone use (*β*=.42, *P*<.001) were important factors of health information seeking, whereas the effect of online information quality was marginal (*β*=–.04, *P*<.001).

**Conclusions:**

Among smartphone users in Asia, health information seeking varies according to individuals’ socioeconomic status, their innovativeness toward technology, and their frequency of smartphone use. Although smartphones widen the digital divide among individuals with different socioeconomic status, they also bridge the divide between countries with varying health expenditures. Smartphones appear to be a particularly useful complement to manage health in developing countries.

## Introduction

### Background

With the development of mobile technology and the spread of its use, smartphones have facilitated several positive health-related outcomes, including the increased provision of psychological interventions [1], improved access to health services, and reductions in various forms of social inequality [2] in populations worldwide. In that context, mobile health (mHealth) refers to public health or medical practices that involve using mobile information communication technologies (ICTs) to seek out health information, communicate with health care professionals, and monitor personal health [3]. Since mHealth provides individuals with ubiquitous access to timely health services at low cost, it can play a major role in bridging inequalities in digital access to health care services [4,5].

In particular, the act of seeking health-related information online has received substantial research attention, but mainly in western countries. For example, analyses of the data from several surveys on the topic administered in the United States and Germany [6,7] have provided valuable insights into the determinants and outcomes of such information-seeking behavior in those populations. By contrast, research investigating such behavior in Asian countries has been sorely limited, especially with respect to health information seeking on smartphones [8].

To address this gap in the literature, we designed this study to investigate the factors contributing to mHealth information seeking (MHIS) to profile such information seekers in Asia using data from 10 Asian countries. In effect, our comprehensive profiling stands to help service providers understand the mHealth market from the perspective of users. We also sought to examine the extent to which individual- and country-level digital divides exist in MHIS across various Asian countries. To the best of our knowledge, this project represents by far the largest multinational survey on smartphone users in Asia. Thus, its findings are expected to provide theoretical insights into both MHIS and its practical implications, all of which may support efforts to bridge the digital divide in Asia.

### Profiling mHealth Information Seekers

The primary objective of this study was to investigate factors related to MHIS to ultimately profile such information seekers in Asia. Previous research has examined correlates of using mHealth that can be classified into four groups: consumers’ motivations and needs for health information [9,10]; driving factors behind adopting mHealth according to theories about technology and health behavior (eg, technology acceptance model, diffusion of innovation theory, and comprehensive model of information seeking) [8,11,12]; sociodemographic features of mHealth users, including their age, gender, education, and income [13-16]; and boundary conditions (eg, cultural context) of theoretical models applied in examining the use of smartphones for health-related purposes [17-19].

Zhao et al [11] recently performed a meta-analysis of 35 empirical studies on mHealth, the overall results of which diverged depending on the context studied and the characteristics of the sample. Their meta-analysis also revealed that the findings of most studies have been based on data from small samples in single-culture contexts, whereas few studies have used multinational data from diverse cultural contexts. Moreover, Wang et al [8] found that technological factors were important yet neglected factors in determining individuals’ online health information–seeking behaviors. Thus, in response to these gaps, we profiled mHealth information seekers in Asia according to their sociodemographic characteristics as well as perceptions of technology.

Parallel to the rise of mHealth technologies, discussions about the quality of health information available online have also intensified. From the perspective of information systems, a high level of *information quality*—defined as the credibility and reliability of information in terms of relevance, sufficiency, accuracy, and timeliness [20]—promotes the use of information systems [21]. At the same time, studies have highlighted users’ concerns with the quality of health information available online, which could hinder online information seeking about health-related topics [22]. Nevertheless, few studies have examined how information quality affects the behavior of MHIS [8].

Against this trend, research has suggested that the adoption of new technology may be primarily driven by how individuals perceive its innovativeness [23]. According to the diffusion of innovation theory, *technology innovativeness*, defined as the degree to which an individual perceives a technological device to be innovative and is thus willing to experiment with using it, is an important factor for adopting technology [24]. Recently, technology innovativeness was also identified as a significant determinant of the intention to use mHealth [25]. In light of these findings, we aimed to scrutinize the effect of online information quality and technology innovativeness on MHIS.

### The Digital Divide and Seeking Health Information on Smartphones

The ability to seek health-related information online has been found to enable individuals to make informed decisions about their health, provide individuals in need with disease-related social support, and help patients adhere to their medication and treatment regimens [8,19]. Nevertheless, extensive research has also revealed that not all individuals benefit equally from assessing and consuming health information online [6,7,26,27]. A critical reason for this inequality is the so-called “digital divide,” a phenomenon related not only to internet access but also to the existence of a gap between people who can and cannot effectively use new communication tools (eg, smartphones) or comprehend new information [28]. Past findings have additionally suggested that smartphones can act either as a bridge or as a barrier for people in assessing health-related information, depending on their socioeconomic status (SES) [6,29-31].

Thus, in a final contribution to the literature, we also tested the digital divide in MHIS at the individual as well as national levels in Asia. To date, scholars have suggested that the global digital divide has narrowed the most for mobile phone use, likely because many developing countries have simply stopped using fixed-line communication as their access to technology has advanced [32]. Therefore, we expected that with smartphones, residents in less developed countries can easily access the internet and health information, which may in turn reduce the inequality in MHIS in those countries.

## Methods

### Procedure and Participants

For our sample, 9086 adults in 10 Asian countries (China, India, Indonesia, Thailand, the Philippines, Malaysia, South Korea, Japan, Vietnam, and Singapore) were recruited in 2016. In line with the procedures of cross-cultural research [33,34], the data were collected from major cities in all countries using synchronous data collection between June 2016 and October 2016. The questionnaire developed for this purpose was translated into each country’s dominant language using standard translation and back-translation, after which it was distributed in each country through an online survey company. To participate, individuals had to have a smartphone and be between 18 and 55 years of age. The total sample comprised at least 800 respondents from each country who were evenly distributed by age and gender in order to fully capture the situation of each stratum in the population. Given these methods, the Institutional Review Board at Nanyang Technological University approved the study.

The participants were adult smartphone users from 10 Asian countries: mainland China (n=1238), India (n=1238), Indonesia (n=824), Thailand (n=821), the Philippines (n=843), Malaysia (n=837), South Korea (n=858), Japan (n=804), Vietnam (n=809), and Singapore (n=814). In total, 9086 smartphone users were recruited. Participants ranged in age from 18 to 55 years, and the majority were women. Nearly three-quarters of the participants had completed college, university, and/or graduate school, and more than half were married and had at least one child. Approximately 88% of participants were employed or self-employed. Detailed descriptive statistics of the sample are given in Table 1.

**Table 1:** Demographic characteristics of the sample.

Characteristic		All participants (N=9086)	Frequent seekers^a^ (n=6508)	
**Gender, n (%)**				
	Man	4716 (51.90)		3292 (50.58)
	Woman	4370 (48.10)		3216 (49.42)
Age (years), mean (SD)		34.3 (9.11)	33.7 (8.80)	
**Marital status, n (%)**				
	Married	5369 (59.09)		3889 (59.76)
	Single, divorced, separated, or widowed	3717 (40.91)		2619 (40.24)
**Have child(ren), n (%)**				
	No children	3924 (43.19)		2526 (38.81)
	At least one child	5162 (56.81)		3982 (61.19)
**Level of education, n (%)**				
	High school or less	2580 (28.40)		1686 (25.91)
	College or university	5101 (56.14)		3755 (57.70)
	Graduate school or more	1405 (15.46)		1067 (16.40)
**Employment status, n (%)**				
	Employed	7971 (87.73)		5808 (89.24)
	Unemployed, homemaker, or retired	1115 (12.27)		700 (10.76)
Smartphone use, mean (SD)		4.87 (1.17)	5.16 (1.01)	
Concern with online information quality, mean (SD)		5.15 (1.18)	5.23 (1.15)	
Technology innovativeness, mean (SD)		4.42 (1.46)	4.68 (1.34)	

^a^“Frequent seekers” reported seeking health information on their smartphones at least a few times per month.

### Measures

#### Quantifying MHIS

Participants reported how frequently they used their smartphones to seek information about health and medical issues on a 5-point scale (1=*never*, 2=*rarely* [ie, once per month], 3=*sometimes* [ie, a few times per month], 4=*often* [ie, a few times per week], 5=*always* [ie, daily]). This item was adopted to measure participants’ MHIS (mean 3.17, SD 1.23).

#### Demographics

Participants reported their gender (0=*man*, 1=*woman*), age, marital status (1=*married*, 0=*single, divorced, separated, or widowed*), and parental status (1=*have child(ren)*, 0=*no children*).

#### Objective SES

Level of education, employment status, and monthly household income were employed as indicators of objective SES [35]. Participants were asked to report their level of education (0=*high school or less*, 1=*college or university*, 2=*graduate school or more*), employment status (0=*unemployed*, 1=*employed*), and monthly income. Given that income levels vary across countries, we standardized the reported monthly income within each country to enable comparisons and analyses across the 10 countries.

#### Subjective SES

A scale for measuring subjective SES was employed [36], in which participants were asked to rate their perceptions of their SES on a 10-rung hierarchical scale. The bottom of the scale, where the score was 1, represented participants who perceived themselves as having the least wealth, the least education, and the least-respected jobs, or no job whatsoever, compared with those of others. The top of the scale, where the score was 10, represented participants who perceived themselves as having the most wealth, the most education, and the most respected jobs relative to others. Participants were asked to place an “X” on the rung of the scale that they believed best reflected their SES (mean 5.84, SD 1.78).

#### Technological Factors

Technological factors included participants’ self-reported perceptions of technology and frequency of smartphone use. Items were adapted from previous studies that involved the measurement of similar concepts [37,38].

Perceived online information quality was assessed on a 7-point Likert scale from 1 (*strongly disagree*) to 7 (*strongly agree*). Participants rated to what extent they agree with the following two items: “In general, there is less control over the quality of the content posted online” and “There is a lot of fake news online these days.” The answers were recoded such that a larger number indicated a higher perceived quality of information online (mean 5.16, SD 1.22; *r*=0.59).

Technology innovativeness was assessed on a 7-point Likert scale from 1 (*strongly disagree*) to 7 (*strongly agree*). Participants indicated their technology innovativeness on the following three items: “Your friends describe you as ‘into the latest technology’,” “You often purchase new technology before your friends,” and “You consider yourself technologically sophisticated” (mean 4.43, SD 1.40; Cronbach *α*=.91).

The frequency of smartphone use was determined according to participants reporting how often they used their smartphones for the following activities in the past year: (1) keeping up to date with news and information, (2) using social networking sites or apps, (3) using electronic banking, and (4) playing games or watching entertaining videos. The answers were recorded on a 5-point Likert scale where 1=*never*, 2=*rarely* (once a month), 3=*sometimes* (few times a month), 4=*often* (few times a week), and 5=*always* (daily). The four items were averaged on a scale of the frequency of smartphone use (mean 3.91, SD 0.78).

#### Country-Level Digital Divide

The country-level digital divide was measured using the ICT Development Index (IDI) from the International Telecommunication Union database of development indicators [39], which indicates the level of the development of ICT infrastructure in a given country. The IDI generates a score from 1 to 10, and of the countries sampled, India scored the lowest (ie, 3.03) and South Korea scored the highest (ie, 8.85). Economic inequality at the country level was measured using the Gini index from the database of World Bank Development Indicators [40]. The Gini index generally ranges from 0% (*perfect equality*) to 100% (*perfect inequality*); of the countries sampled, the Philippines scored the highest (ie, 44.4%), whereas South Korea scored the lowest (ie, 31.6%). Health inequality at the country level was measured by each country’s current health expenditure (CHE) per capita based on the global health expenditure database maintained by the World Health Organization [41], which is reported in purchasing power parity, ranging from 253 in India to 4563 in Japan.

### Data Analysis

Multilevel linear regression models were constructed to determine the effect of individual-level characteristics and country-level inequalities in MHIS, after which analyses were performed using the lme4 package in R, an open-source program for statistical analysis. The model-building process involved three steps: (1) a univariate analysis of each variable using appropriate statistical tests (eg*,* *t* test, analysis of variance, or Pearson correlation), (2) a model including any variables whose univariate test had a *P* value less than .25, and (3) a two-level linear model with variances specified at the individual level and country level. In this paper, the results of multilevel linear regression analysis are presented in terms of their standardized *β* coefficients and model statistics. A two-sided *P* value of less than .05 for all tests was considered to be statistically significant.

## Results

### Descriptive Analysis

The sociodemographic characteristics of the participants are shown in Table 1. Of the 9086 smartphone users sampled, only 996 (10.96%) had never sought health-related information on their smartphones. By contrast, 71.63% (n=6508) reported seeking such information on their smartphones at least a few times per month. Other than seeking health-related information, most participants reported regularly using smartphones to keep themselves informed of the latest news and information (8650/9086, 95.20%), build and maintain social networks (8432/9086, 92.80%), use electronic banking (7578/9086, 83.40%), play video games (7196/9086, 79.20%), and watch entertaining videos (7196/9086, 79.20%). Generally, the participants considered themselves to be interested in technological innovation and new technology (mean 4.42, SD 1.46). Regarding their concerns over the quality of online information, more than half agreed (Likert scale>4) that there is less control over the quality of online content than content from other media outlets (6108/9086, 67.22%) and that there is a lot of fake news online (6766/9086, 74.47%).

### Individual-Level Analyses

In the univariate analyses, the frequency of using smartphones to find health information was significantly higher among participants who were women (*t*_9085_=4.00, *P*<.001), married (*t*_9085_=–3.25, *P*=.01), employed (*t*_9085_=–9.06, *P*<.001), parents (*t*_9085_=–16.83, *P*<.001), and/or had a high monthly household income level (*r*=0.06, *P*<.001) and a high level of education (*F*_2,9084_=58.0, *P*<.001). Moreover, the frequency of using smartphones to seek health information was positively associated with the level of subjective SES (*r*=0.29, *P*<.001) but was negatively associated with age (*r*=–0.09, *P*<.001).

In terms of technology-related factors, the frequency of using smartphones to search for health information was significantly and positively associated with the frequency of smartphone use in general (*r*=0.51, *P*<.001). Additionally, concern about online information quality (*r*=0.14, *P*<.001) was significantly and positively related to seeking health information on smartphones.

The results of the multilevel regression analysis substantially confirmed the findings of the univariate analyses (Table 2). For individual-level characteristics, individuals who frequently sought health-related information using their smartphones were more likely to be women, to be employed, to have at least one child, and to perceive themselves as having a high SES. At the same time, age, household income, and marital status were not significantly related to MHIS in the regression model. Frequencies of smartphone use and technology innovativeness were positively related to seeking health information. However, the relationship between concern over online information quality and MHIS became negative with the presence of other factors. Model statistics are summarized in Table 3.

**Table 2:** Multilevel regression analyses of mobile health information seeking.

Variable			Model 1	Model 2		Model 3	
				*β* (SE)	*P* value	*β* (SE)	*P* value
Intercept			–.02 (.15)	.06 (.07)	.09	–.08 (.05)	.07
**Individual level**							
	**Gender**						
		Woman	—^a^	Reference	Reference	Reference	Reference
		Man	—	–.13 (.02)	<.001	–.13 (.02)	<.001
	Age		—	–.01 (.01)	.13	.01 (.01)	.11
	**Marital status**						
		Single, divorced, separated, or widowed	—	Reference	Reference	Reference	Reference
		Married	—	-.004 (.02)	.42	–.004 (.02)	.41
	**Education**						
		High school or less	—	Reference	Reference	Reference	Reference
		College or university	—	.02 (.03)	.14	.02 (.02)	.13
		Graduate school or more	—	.08 (.02)	.004	.07 (.03)	.009
	Monthly income		—	-.02 (.01)	.06	-.02 (.01)	.06
	Subjective SES^b^		—	.08 (.01)	<.001	.08 (.01)	<.001
	**Employment**						
		Unemployed	—	Reference	Reference	Reference	Reference
		Employed	—	.08 (.02)	<.001	.08 (.02)	.002
	**Parental status**						
		No children	—	Reference	Reference	Reference	Reference
		Have at least one child	—	.16 (.02)	<.001	.16 (.02)	<.001
	Concern with online information quality		—	–.04 (.01)	<.001	–.04 (.01)	<.001
	Technology innovativeness		—	.11 (.01)	<.001	.10 (.01)	<.001
	Frequency of smartphone use		—	.42 (.01)	<.001	.42 (.01)	<.001
**Country level**							
	IDI^c^		—	—	—	.06 (.09)	.26
	CHE^d^		—	—	—	–.19 (.09)	.02
	GINI^e^		—	—	—	–.03 (.05)	.22

^a^Not included in model.

^b^SES: socioeconomic status.

^c^IDI: Information Communications Technology Development Index.

^d^CHE: current health expenditure per capita (purchasing power parity, 2017).

^e^GINI: Gini index (World Bank estimate).

**Table 3:** Statistics for the multivariate regression models.

Model statistic	Model 1	Model 2	Model 3
Level 1 variance (SD)	0.88 (0.94)	0.66 (0.18)	0.66 (0.82)
Level 2 variance (SD)	0.15 (0.38)	0.03 (0.18)	0.02 (0.13)
Intraclass coefficient	N/A^a^	N/A	0.14
Log-likelihood	24685.7	22167.3	22160.1

^a^N/A: not applicable.

### Country-Level Analyses

Table 4 presents the overall descriptive analysis at the country level. We found that the frequency of seeking health information using smartphones was the highest in Vietnam (mean 4.57, SD 1.83), followed by Indonesia (mean 4.31, SD 1.63), India (mean 4.06, SD 1.73), China (mean 4.00, SD 1.60), the Philippines (mean 3.96, SD 1.62), Thailand (mean 3.94, SD 1.72), Malaysia (mean 3.53, SD 1.68), South Korea (mean 3.48, SD 1.72), Singapore (mean 3.10, SD 1.54), and Japan (mean 2.29, SD 1.46). The distribution of overall smartphone use across countries showed a similar pattern to that of using such technology to seek health information. People in Vietnam (mean 5.05, SD 1.15), China (mean 5.03, SD 0.94), India (mean 4.90, SD 1.06), Thailand (mean 4.85, SD 1.05), Indonesia (mean 4.80, SD 1.00), and the Philippines (mean 4.76, SD 0.93) reported a higher frequency of using their smartphones for news and information, electronic banking, social networking, and entertainment than people in Malaysia (mean 4.44, SD 1.06), South Korea (mean 4.40, SD 1.08), Singapore (mean 4.18, SD 1.06), and Japan (mean 3.61, SD 1.20).

**Figure 1:**
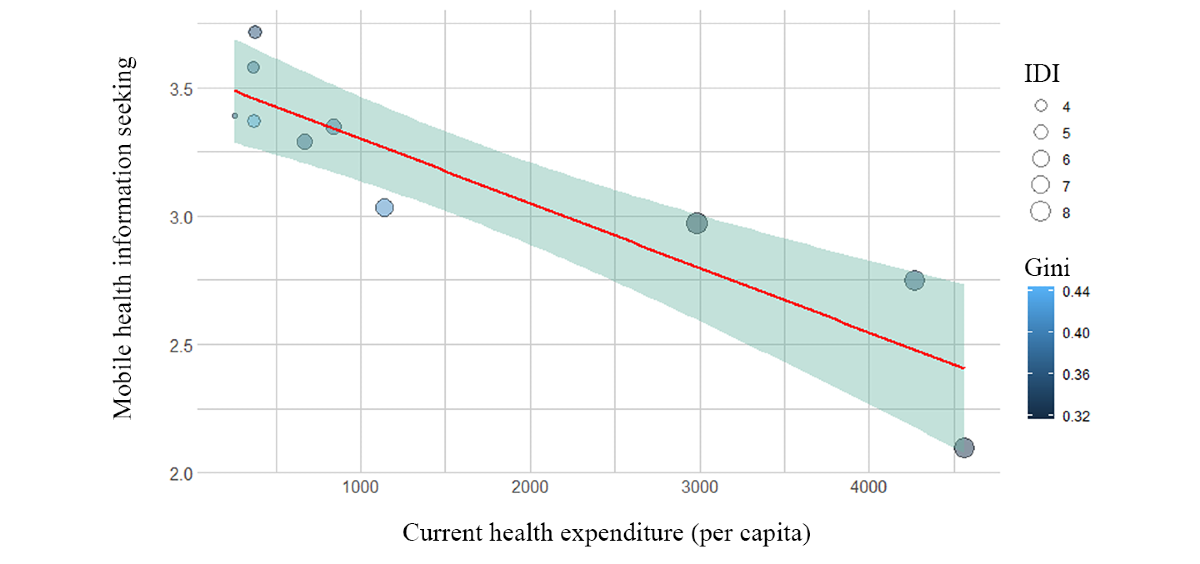
Current health expenditure (per capita) and mobile health information seeking in 10 Asian countries. The size of the nodes represents the Information Communications Technology Development Index (IDI), and the shading of the nodes represents the Gini index of the countries.

Figure 1 shows a scatterplot of the relationship between CHE, IDI, the Gini index, and MHIS among the 10 Asian countries. All three factors of inequality at the country level were highly related to information-seeking behavior. Countries with a low Gini index, low IDI, and low CHE were more likely to rely on smartphones as a source for their health information. However, the multilevel regression analysis (Table 2) showed that CHE was the only significant factor related to individuals’ MHIS. Economic inequality and IDI were not significantly associated with MHIS in the regression model.

**Table 4:** Country-level statistics.

Country	Sample, n	MHIS^a^ (SD)	General smartphone use (SD)	COIQ^b^ (SD)	IDI^c^	CHE^d^	GINI^e^
China	1238	3.34 (1.11)	4.11 (0.67)	4.85 (1.12)	5.60	841.1	38.6
India	1238	3.39 (1.19)	4.09 (0.71)	5.36 (1.20)	3.03	253.3	35.7
Indonesia	824	3.58 (1.11)	4.03 (0.70)	5.31 (1.11)	4.33	367.9	38.1
Japan	804	2.09 (1.10)	3.11 (0.93)	4.28 (1.43)	8.43	4563	32.1
South Korea	858	2.97 (1.21)	3.68 (0.79)	4.94 (1.19)	8.85	2980	31.6
Malaysia	837	3.03 (1.18)	3.82 (0.74)	5.37 (1.06)	6.38	1139	41.0
Philippines	843	3.37 (1.12)	4.10 (0.64)	5.55 (1.10)	4.67	371.7	44.4
Singapore	814	2.75 (1.11)	3.64 (0.78)	5.23 (1.00)	8.05	4270	35.6
Thailand	821	3.29 (1.20)	4.10 (0.71)	5.35 (1.12)	5.67	670.9	36.5
Vietnam	809	3.72 (1.20)	4.18 (0.71)	5.46 (1.33)	4.43	375.6	35.5
All	9086	3.17 (1.23)	3.91 (0.80)	5.16 (1.22)	5.94	1583	36.9

^a^MHIS: mobile health information seeking.

^b^COIQ: concern with online information quality.

^c^IDI: Information Communications Technology Development Index.

^d^CHE: current health expenditure per capita (purchasing power parity, 2017).

^e^GINI: Gini index (World Bank estimate).

## Discussion

### Principal Findings

Understanding how individual- and country-level differences in MHIS are affected by socioeconomic conditions is important for developing and evaluating public health policy. Studies have suggested that the digital divide is a serious barrier that largely contributes to health inequality [42]. In our study, we extended these findings by examining MHIS in Asia and the digital divide associated with such behavior at both the individual and country levels. Based on a sample of 9068 participants from 10 Asian countries, our results suggest that the act of seeking health information with smartphones varies according to individuals’ SES, perceptions of technology, and country of residence. It seems that smartphone technology widens the digital divide throughout the socioeconomic structure of society, such that individuals who are of higher education or subjective SES, married, parents, and/or employed are more likely to use smartphones to seek health information. However, smartphones also bridge the gap between countries to some extent. Individuals from countries with lower expenditures in health are more likely to use smartphones to seek health information. The multilevel digital divide documented in our study has practical implications for public health professionals.

### Profiling Mobile Health Information Seekers in Asia

Being a woman has consistently predicted increased activity in seeking health information online [13-16]. Women tend to use smartphones for obtaining health information more often than men owing to their higher engagement in health-related activities for themselves and their family members. In that light, our study extends past findings to the Asian context. Interestingly, although age has been emphasized as an important factor of social division in previous studies [6], its effect was not significant in our regression models, possibly due to the nonlinear relationship between age and MHIS. Younger and older generations emerged as being more likely to use smartphones to seek health information than middle-aged individuals. This may reflect the fact that the younger generation is more familiar and comfortable with using smartphones to meet their everyday needs, including health-related needs, whereas the older generation has a strong motivation and need to seek health information from any source.

Our findings also highlight the important role of technological factors in explaining MHIS. The perceived innovativeness of technology was a primary factor for accepting new technology, including in relation to mHealth. Information quality surfaced as another factor related to seeking health information, because the information involved in these practices is highly personal and thus sensitive. However, previous studies on mHealth have not sufficiently investigated the role of these technological factors [8]. Future studies aiming for a comprehensive understanding of MHIS should thus include the factors of technology innovativeness and information quality.

### Individual-Level Digital Divide

Our study confirmed that individuals who are of higher education or social status, married, parents, and/or employed are more likely to use smartphones to seek health information. Therefore, our results extend previous findings to the Asian context. The digital divide in health refers to inequalities not only in internet access, mobile technology, and social media but also in the ability to comprehend the information found online. Although access to the internet or smartphones is now ubiquitous in most Asian countries, a second level of socioeconomic inequalities such as different levels of education and SES affect individuals’ ability to seek and comprehend online information [8,28]. Therefore, future studies should focus on this second level of the digital divide and its influence on mHealth across various social groups. Public health efforts attempting to leverage the power of mobile technology should also adopt different strategies to avoid inequalities across social structures. For instance, online communication–based interventions should better investigate and address issues pertaining to eHealth literacy, including the ability to seek, find, understand, and appraise health information from electronic sources, so as to reduce inequalities in communication across different socioeconomic groups.

### Country-Level Digital Divide

At the country level, we found that participants from countries that spend less on health per capita were more likely to rely on smartphones as a source for health information. This result stresses that smartphones in developing countries, including China, India, the Philippines, Indonesia, Vietnam, and Thailand, may function as a tool for managing daily activities, including seeking health information. Considering that these Asian countries have a relatively high mobile internet penetration rate [43], our results provide evidence that smartphones act as tools that can bridge health inequalities between countries. In developing countries where health or digital resources are limited, mobile technology may also help individuals to access information about health as well as manage their health. Whereas previous studies investigating the digital divide in access to health information and technology have focused on the socioeconomic characteristics, internet access, and information literacy of individuals, our study examined the digital divide at both the individual and country levels. Therefore, this study adds a new dimension for understanding the digital divide in mHealth.

### Strengths and Limitations

The strengths of our study include its large sample, and the collection of data on individuals’ smartphone use and perceptions in several Asian countries. A previous meta-analysis suggested that studies about information-seeking behavior with mobile technology in Asia with large samples have been lacking [8]. Thus, our study has filled this gap through administering by far the largest multinational survey on smartphone users in Asia. Another strength was that two levels of the digital divide were examined, which furnishes considerable knowledge about the digital divide in MHIS.

Nevertheless, the limitations of our study should be noted when generalizing the findings. First, our sample was not representative. Our participants were smartphone owners who live in urban areas in each country sampled. Despite variance in our participants’ SES, their relatively high SES may limit the generalizability of the findings. Second, we only examined the role of socioeconomic factors and technology perceptions on MHIS. Other psychological-related factors (eg, attitude, self-efficacy, perceived risk, worry, and anxiety) should also be taken into consideration in future studies with the aim of forming a comprehensive understanding of such phenomena [8]. Finally, information quality has drawn great attention from both academia and industry because of prevalent misinformation online [44]. Although our study found a marginal effect of information quality, future studies aiming to gain a nuanced understanding of such a phenomenon could examine the multidimensional nature of information quality or the reciprocal relationship between perceived online information quality and information seeking. 

### Conclusion

Among smartphone users in Asia, seeking health information on mobile devices varies according to the users’ SES, perceptions of technology and information, and their governments’ health expenditures, but not in accordance with the ICT divide or economic inequality at the country level. These findings suggest that although smartphones represent a readily available source of health information, they can also create inequalities in the access to health information among different socioeconomic classes of society. At the same time, the findings imply that smartphones are widely accepted as a tool for daily activities and communication in developing areas in Asia. In that light, mobile technology appears to be a particularly useful complement for the management of health in developing countries.

## Abbreviations

CHE: current health expenditure
ICT: information communication technology
IDI: Information Communication Technology Development Index
mHealth: mobile health
MHIS: mobile health information seeking
SES: socioeconomic status
